# Evaluation of Hologic LOCalizer™ RFID Tags for Preoperative Localization of Breast Lesions: A Single-Center Experience

**DOI:** 10.3390/diagnostics15060746

**Published:** 2025-03-17

**Authors:** Charlotte Munday, Anmol Malhotra, Sawsan Taif, Adeola Omotade, Arathi Menon, Kefah Mokbel

**Affiliations:** 1 The Royal Free Hospital, London NW3 2QG, UK; anmolmalhotra@nhs.net; 2 London Breast Institute, London W1G 9QP, UK; 3 Royal Free London NHS Foundation Trust, London NW3 2QG, UK; s.abuflayeh@nhs.net (S.T.); a.menon@nhs.net (A.M.); 4 Whittington Health NHS Trust, London N19 5NF, UK; adeola.omotade@nhs.net

**Keywords:** breast cancer, non-palpable breast lesions, wire-free localization, RFID tags, LOCalizer™, preoperative localization

## Abstract

**Background**: The increasing detection of non-palpable breast lesions necessitates accurate preoperative localization to ensure complete excision while preserving healthy tissue and optimizing cosmetic outcomes. Traditional wire-guided localization (WL) has been the gold standard; however, it has several drawbacks, including patient discomfort and scheduling challenges. This study evaluates the accuracy and feasibility of radiofrequency identification (RFID) tag localization using the Hologic LOCalizer™ system as an alternative technique. **Methods**: This retrospective study included 258 consecutive patients who underwent image-guided RFID tag localization from March 2021 to February 2023 from a single-center London breast unit. The primary outcome measured was the accuracy of RFID tag placement, defined as within 10 mm of the target lesion on post-clip mammograms. Secondary outcomes included type and size of lesions, re-excision rates, review of post-operative specimen radiographs, and patient demographics. **Results**: A total of 297 RFID tags were placed, with 95.6% accurately positioned within the target range. The median target size was 29 mm, with the most common abnormalities being mass lesions (64%). Among the 13 inaccurately placed RFID tags (4.4%), all were identified preoperatively, with two requiring additional wire placements. RFID tags were successfully identified in 92% of specimen radiographs, and 8% of patients required re-excision due to positive or close margins. Notably, patients with multiple RFID tags showed a higher incidence of re-excision. **Conclusions**: The LOCalizer™ RFID system demonstrated a high accuracy rate for preoperative localization of breast lesions, presenting a viable alternative to WL. This technique improves surgical scheduling flexibility and enhances patient comfort. Comparative studies with other wire-free localization technologies, such as magnetic seeds and radar reflectors, are needed to determine the optimal approach for clinical practice.

## 1. Introduction

Advances in imaging technology and the widespread implementation of screening mammography have significantly improved the early detection of non-palpable breast lesions, leading to increased diagnosis rates of early-stage breast cancer [1]. The ability to detect these lesions before they become clinically evident has contributed to a shift in breast cancer management, with a strong emphasis on breast-conserving surgery (BCS) as the preferred treatment approach when oncologically appropriate [2,3,4]. Multiple randomized controlled trials have demonstrated that BCS, when combined with appropriate adjuvant therapy, provides equivalent survival outcomes to mastectomy while preserving the natural breast contour, thereby improving patient satisfaction and quality of life [2,3,4]. 

Given the widespread adoption of BCS, precise preoperative localization techniques are essential to ensure complete tumor removal while minimizing excision of healthy breast tissue and optimizing cosmetic outcomes [1]. Wire-guided localization (WL) has long been the gold standard for marking non-palpable breast lesions prior to surgical excision [5]. However, despite its efficacy, WL presents several logistical and technical challenges, including patient discomfort, risk of wire migration or dislodgement, and the necessity for same-day wire placement, which imposes significant strain on healthcare resources by requiring close coordination between radiology and surgery teams [1]. 

To overcome these limitations, various wire-free localization techniques have emerged, offering increased flexibility in scheduling and improved patient comfort. These include radioactive seed localization (RSL), non-radioactive radar localization (SAVI SCOUT), magnetic seed localization (Magseed), and RFID [1]. 

The LOCalizer™ system (Hologic, Marlborough, MA, USA) is based on ubiquitous RFID technology, offering a wire-free alternative for the preoperative localization of non-palpable breast lesions. It consists of an RFID tag with a unique identification number preloaded in a needle applicator, a surgical probe with an 8 mm tip, and a portable handheld reader. The RFID tag itself is small, measuring approximately 11 mm in length and 2 mm in diameter, allowing for minimal tissue disruption upon placement. Notably, the RFID tag includes a polypropylene cap designed to prevent migration within the breast tissue, ensuring stable localization until and during surgery. The tracer chip is deployed using a 12 G introducer, ensuring precise placement within the breast tissue. Once implanted, the RFID tag emits a radiofrequency signal that is detected by the surgical probe, which is roughly the size of a pencil. The reader, which is lightweight and portable, displays real-time feedback on the distance to the RFID tag in millimeters, which, along with the RFID tag’s unique ID number, enables accurate localization. Unlike some other localization methods, the LOCalizer™ RFID tag does not require radiation or an external energy source to function, enhancing safety and logistical flexibility. The device is certified for long-term implantation, allowing for placement days or even weeks before surgery without compromising accuracy. Arguably, utilizing a widespread and well-understood technology such as RFID makes LOCalizer™ a robust and reliable choice for breast lesion localization, streamlining surgical workflows and improving patient comfort [5]. 

The purpose of this retrospective study was to assess the accuracy of preoperative RFID tag localization using the LOCalizer™ system and to document our initial experience with this technique in our single-center London breast unit. RFID tag localization using the LOCalizer™ system had been incorporated at our unit and approved locally as our method of choice for wire-free localization. Thus, this study aimed to evaluate our early experience and accuracy of this new technique at a local level. RFID tag placement accuracy was defined as being within 10 mm of the edge of the target lesion (Table 1). Our benchmark of >95% of RFID tags being within 10 mm of the lesion was modeled on the NHS Breast Screening Programme’s standard, which considers >95% of localization wires within this range as acceptable [6]. 

Table 1 summarises the primary and secondary objectives and outcomes of the study.

## 2. Materials and Methods

The cohort for this retrospective study included the first consecutive patients undergoing image-guided localization with RFID tags (LOCalizer™, Hologic, Marlborough, MA, USA) prior to surgical excision from March 2021 to February 2023, comprising a total of 258 patients. Patients included in the study were from our single-center London breast unit, and all subsequent breast surgeries were performed at this institution. Imaging and medical records were reviewed to evaluate the number of RFID tags placed and the RFID tag placement accuracy rate, defined as within 10 mm of the edge of the target lesion. Data collected also included patient age, the type of lesion/abnormality being targeted (e.g., impalpable lesions, masses, asymmetric densities, distortions, and microcalcifications [MCC]), lesion size (if applicable), and localization indication. The postoperative specimen pathology radiograph was reviewed to confirm the presence of the RFID tag. Post-surgical histopathology reports were assessed to calculate the number of patients requiring re-excision. 

**Figure 1:**
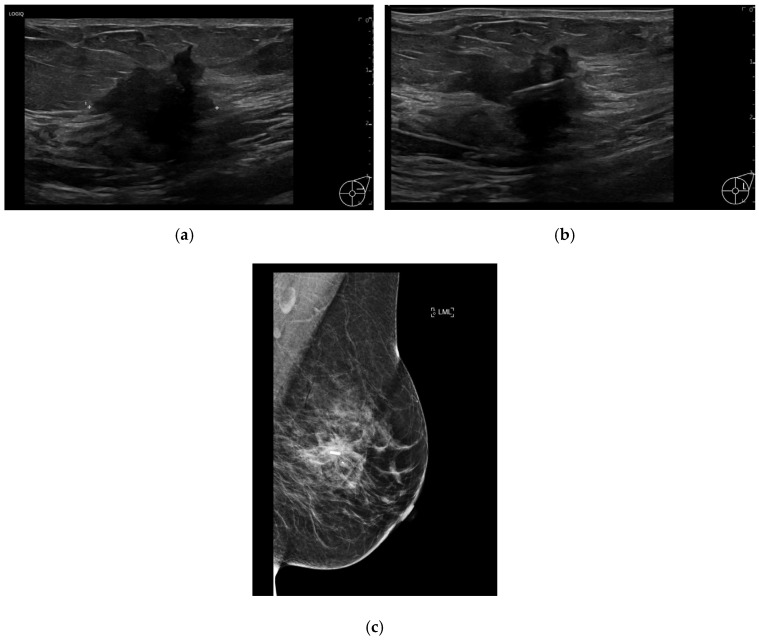
(**a**) Ultrasound of the left breast demonstrates a 25 mm hypoechoic, irregular lesion within the upper outer quadrant, which was also noted to have internal vascularity. Biopsy results yielded invasive ductal carcinoma (grade 3). (**b**) Ultrasound image obtained during localization shows a RFID tag deployed within the center of the lesion. (**c**) Mediolateral oblique view post-localization mammogram of the left breast shows successful RFID tag placement within the center of the lesion.

**Figure 2:**
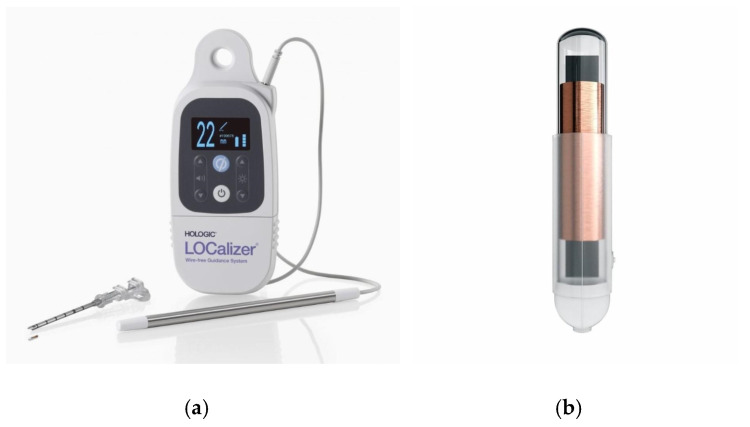
(**a**) Radiofrequency RFID tag localization system (LOCalizer™, Hologic) handheld reader device attached to the surgical probe and adjacent to the RFID tag device. (**b**) Radiofrequency identification RFID tag (10.6 mm × 2 mm in dimension) with a polypropylene cap helping to prevent migration within breast tissue.

The RFID tags were inserted by either a consultant breast radiologist or a breast radiology trainee under ultrasound or mammographic guidance (Figure 1a–c). RFID tag insertion was performed during a dedicated appointment prior to surgery, allowing for preoperative planning and minimizing logistical challenges on the day of surgery. The LOCalizer™ system comprises a unique RFID tag, which measures approximately 11 mm in length and 2 mm in diameter, deployed using a 12-gauge introducer. Each RFID tag includes a polypropylene cap designed to prevent migration within the breast tissue, ensuring stability throughout the preoperative period. After RFID tag insertion, a handheld portable reader displays the distance to the RFID tag in millimeters, which is particularly valuable when deploying more than one RFID tag (e.g., in cases of bracketing or multifocal disease). The system also assigns an individual ID RFID tag number, which is recorded in the patient’s medical notes (Figure 2a,b), facilitating accurate intraoperative retrieval. 

**Figure 3:**
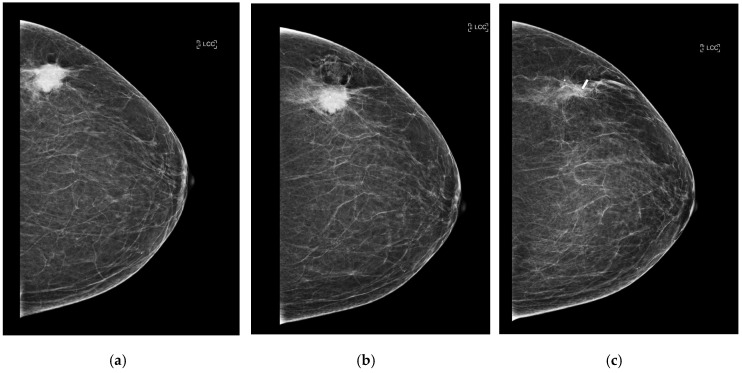
(**a**) Craniocaudal (CC) view of the left breast of predominately fat density (BI-RADS A). There is a 27 mm malignant-appearing mass in the upper portion of the left breast. (**b**) CC post-biopsy mammogram shows the marker clip within the center of the lesion. Following ultrasound-guided core needle biopsy and accurate clip placement, biopsy results yielded invasive ductal carcinoma (grade 3). The patient underwent neo-adjuvant chemotherapy followed by radiofrequency RFID tag localization. (**c**) CC view of the left breast. The known mass within the upper outer quadrant has reduced in size following neo-adjuvant chemotherapy and measures 19 mm (from 27 mm initially). The post-localization mammogram shows successful RFID tag placement (at the lateral aspect of the lesion).

**Figure 4:**
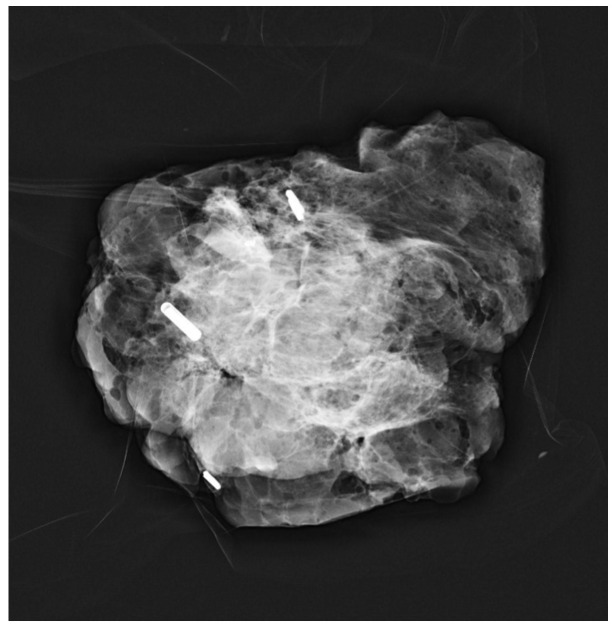
Radiograph of the specimen shows successful retrieval of the mass and RFID tag (note the post biopsy marker is not identified within this specimen however present on another sample).

After placement, standard two-view mammograms were obtained to confirm RFID tag placement. Placement accuracy was recorded by measuring the shortest distance, on either the mediolateral oblique (MLO) or craniocaudal (CC) view, from the RFID tag to the intended target (e.g., target lesion, distortion, MCC) on the post-clip mammograms. Following surgical excision, a specimen radiograph was performed to confirm RFID tag presence and location (Figure 3a–c and Figure 4). Additionally, any cases of RFID tag migration were documented, along with factors such as hematoma formation or lesion characteristics that may have influenced RFID tag positioning. Data collection was conducted in compliance with institutional ethical guidelines, ensuring patient confidentiality and adherence to research standards. 

### Statistics

Confidence intervals (CIs) for proportions were calculated using the Wilson score method without continuity correction, which provides a reliable estimate for small sample sizes. Continuous variables, such as lesion size and patient age, were summarized using medians and interquartile ranges (IQR) due to the non-normal distribution of data. The accuracy of RFID placement was analyzed using descriptive statistics, with categorical variables compared using the chi-square test or Fisher’s exact test as appropriate. For cases requiring re-excision, logistic regression was considered to explore potential predictors, but the sample size was insufficient for robust multivariate modelling. 

## 3. Results

**Figure 5:**
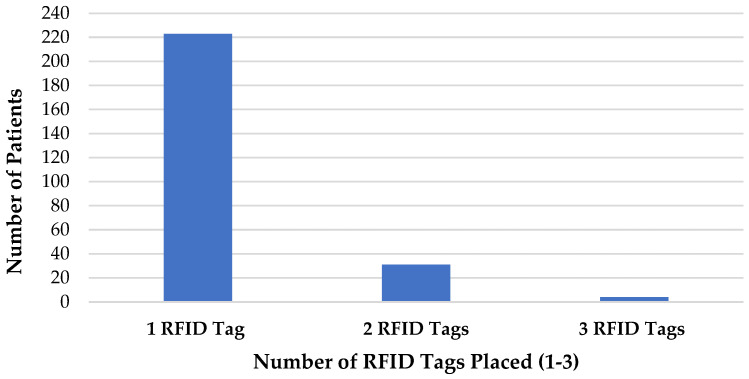
Number of RFID tags placed in the patients involved in the study.

**Figure 6:**
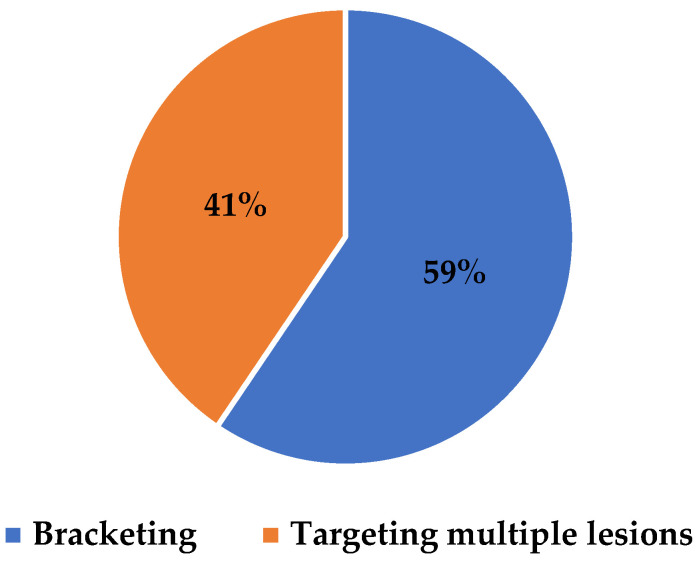
Purpose of the multiple RFID tags placed.

During the study period, a total of 258 patients (mean age 61 years; range 17–91 years) underwent image-guided RFID tag localization. The median number of RFID tags placed per patient was one (range 1–3) (Figure 5). A single RFID tag was placed in 223 patients (86.4%, 95% CI: 81.8–90.1%), while 35 patients (13.6%, 95% CI: 9.9–18.2%) had more than one RFID tag inserted, resulting in a total of 297 RFID tags placed. Among the 35 patients had multiple RFID tags, 4 had 3 RFID tags inserted, and 31 patients had 2 RFID tags inserted, totaling 74 RFID tags. Of these, 44 RFID tags were used for bracketing, and 30 were used for targeting multiple breast lesions (e.g., multifocal or bilateral breast cancers) (Figure 6). In patients where more than one RFID tag was inserted, the multiple RFID tags were all inserted within the same clinic visit. All patients were at the MDT where full work up and complete imaging had been obtained prior to RFID tag localization being performed.

The median size of the target lesion was 29 mm (range 0–57 mm). The distribution of target abnormalities and their size ranges are summarized in Table 2. The types of targeted abnormalities are described in Table 2. Mammographically occult lesions included MRI-detected abnormalities or lesions that demonstrated interval resolution/response to neoadjuvant chemotherapy, where marker clips were subsequently targeted for RFID placement. In cases of microcalcifications (MCC), lesion sizes ranged from single MCC clusters to the full anterior-to-posterior extent of multifocal pathological MCC. 

Technical accuracy of RFID tag placement was achieved in 284/297 RFID tags (95.6%, 95% CI: 92.5–97.7%), defined as being within 10 mm of the target/lesion on post-clip mammograms. Of the 13 RFID tags (4.4%, 95% CI: 2.3–7.5%) inaccurately placed, 9 were inserted under ultrasound guidance, and 4 under stereotactic guidance. Among the nine inaccurately placed RFID tags under ultrasound guidance, three were affected by hematoma formation, impacting RFID tag positioning, and four RFID tags demonstrated migration. As this was a new technique at our institution, it is possible that inexperience contributed to the incorrect placement of the remaining two RFID tags. 

The four RFID tags inaccurately placed under stereotactic guidance showed migration of up to 35 mm from the target. All cases of inaccurately placed RFID tags were identified prior to surgery and discussed with the breast surgeons or during the regional MDT meeting. Two patients required additional wire placements on the day of surgery due to small, focal MCC. 

A total of 273/297 RFID tags (92%, 95% CI: 88.4–94.7%) were present in the specimen radiograph, while 24/297 RFID tags (8%, 95% CI: 5.3–11.6%) were absent. Of the absent RFID tags, 5/24 were placed in patients with more than one RFID tag. Four of these five RFID tags were placed in patients undergoing bracketing (two RFID tags), where the remaining RFID tag was present in the specimen radiograph. The fifth RFID tag was from a patient with multifocal disease where three RFID tags were placed; the other two RFID tags were present in the specimen radiograph. All the patients in which the RFID tags were missing from the specimen radiographs had post-treatment mammograms within one year of their surgery, demonstrating that all the RFID tags had been successfully removed intraoperatively. Theories as to why some of the RFID tags were missing from the specimen radiographs include the RFID tags being dislodged/expelled from the specimen (e.g., when being transferred to the sample pot prior to being sent to histopathology).

Four patients with absent RFID tags in the specimen radiograph required surgical re-excision, and all RFID tags were retrieved. Overall, 21/258 patients (8%, 95% CI: 5.0–11.9%) had positive or close surgical margins necessitating re-excision. Of these, eight patients had more than one RFID tag placed during localization (two patients had three RFID tags, and six patients had two RFID tags). The need for re-excision was higher in patients with extensive microcalcifications or multifocal disease, where multiple RFID tags were used to guide surgery. This suggests the complexity of the disease was likely to have affected the need for re-excision rather than an accurate reflection of RFID tag localization technique.

## 4. Discussion

This study evaluates the use of the LOCalizer™ system for the localization of non-palpable breast lesions, highlighting its accuracy, feasibility, and potential advantages over traditional wire-guided localization (WL). To our knowledge, this is the second largest reported study assessing this technology in a cohort of 258 patients with 297 RFID tags deployed. 

The LOCalizer™ demonstrated a high technical accuracy rate of 95.6% (RFID tag placement within 10 mm of the lesion), comparable to the standards set by WL and consistent with previously published data on RFID localization [5,7]. Importantly, the decoupling of radiological and surgical scheduling offers significant logistical benefits, addressing a key limitation of WL, which requires same-day placement. This scheduling flexibility can improve patient experience and reduce the strain on healthcare systems. Furthermore, the LOCalizer™ reflector’s unique identification number facilitates precise bracketing of large non-palpable breast lesions, enabling accurate surgical targeting. 

Our study’s findings are consistent with those of Malik et al. [8], supporting the effectiveness of RFID technology in localizing non-palpable breast lesions. Malik et al. observed a re-excision rate of 17.27%, with higher rates when the RFID tags were placed outside the lesion (28.94%). In the largest study reported in the literature, Lamb et al. detailed their experience with 1013 RFID tags placed under imaging guidance in 848 patients, achieving a 98.4% successful localization rate and a 15.1% positive surgical margin rate [9]. We identified a re-excision rate of 8% in our cohort, with technical accuracy in 95.6% of RFID placements. While migration of RFID tags or hematoma formation affected placement in a small subset of patients (4.4%), these challenges highlight the importance of operator experience and precision in RFID tag placement. Additionally, our findings demonstrated the utility of RFID tags in more complex scenarios, such as multifocal disease and bracketing, further underscoring the versatility of this technology as a reliable alternative to wire localization. Our re-excision rate is comparable to that reported for other wire-free localization technologies and appears lower than the rates associated with wire localization (WL) [8,9,10]. 

A recent study found that RFID tag localization for non-palpable breast lesions resulted in higher patient satisfaction compared to WL, attributed to greater comfort and reduced anxiety [11]. Patient questionnaires included the evaluation of pain during placement, pain between placement and surgery, stress during placement, and overall satisfaction. These findings are further supported by a study conducted in 2016, which compared patient satisfaction of radioactive seed localization (RSL) with WL [12]. Results showed that patients in the RSL group reported less pain during localization and better global satisfaction than patients in the WL group. We theorize that the sight of the wire coming out of the breast in those patients undergoing WL could be a source of anxiety, impacting pain perception and overall satisfaction (unlike non-wire localization techniques, which are not seen by the patient). Given that wires are inserted on the day of surgery, it is likely patients are also under a high degree of stress, influencing the psychological impact of the experience.

However, our findings also reveal some limitations of the LOCalizer™ system. Migration accounted for the majority of inaccurate placements (8/13 cases), likely due to its smooth casing, learning curve effects, the wide tract created by the 12-gauge introducer needle, and limited anchoring within soft tissue [5,8]. Additionally, the MRI void artifacts (2 cm), although smaller than those caused by Magseed (4 cm), may limit its use in certain patients, particularly those undergoing neoadjuvant systemic therapy and MRI surveillance [5,9]. The use of Magseed, however, necessitates the removal of all metal instruments from the surgical field during dissection, which can hinder the adoption and implementation of this localization technique [9]. Finally, the LOCalizer™ system is not currently licensed for deployment within axillary lymph nodes to facilitate targeted axillary dissection following neoadjuvant systemic therapy (NST). 

The limitations of our study include its retrospective design, which may introduce selection bias, and the single-center setting, which could limit the generalizability of our findings. Further analysis comparing RF RFID tag insertion modalities (ultrasound vs. stereotactic) could provide insights into which techniques are more prone to inaccurate placement. The absence of a direct comparison with wire localization in a controlled setting further limits the ability to comprehensively assess the advantages of RFID technology. 

A number of advancements could enhance the performance and versatility of the LOCalizer™ system. One key area for improvement is the reduction in MRI-related void signals, which can interfere with imaging quality. These void signals are notably larger than those produced by radioactive seed localization (RSL) and SAVI SCOUT (less than 5 mm) [10]. Optimizing the RFID tag’s material composition or adjusting its resonance frequency could help minimize MRI artifacts, improving its compatibility for preoperative planning in patients requiring MRI-guided localization. The development of an MRI-compatible deployment system would also expand the clinical utility of the LOCalizer™ in settings where MRI is the preferred imaging modality. Additionally, refining the RFID tag design to make it even smaller could enhance patient comfort and further reduce the risk of migration, particularly in dense breast tissue. Another potential modification involves using a broader gauge needle for deployment, which could facilitate smoother insertion while minimizing tissue disruption and reducing the likelihood of RFID tag movement. To address migration, modifications to the RFID tag’s surface texture or the addition of bioresorbable anchoring elements could enhance tissue integration and stability. The current polypropylene cap could be redesigned with microbarbs or a porous structure to encourage fibrosis around the RFID tag, securing it more effectively in place. Another potential approach is the use of a hydrogel coating that expands slightly upon implantation, providing additional resistance to displacement. Furthermore, obtaining regulatory approval for using RFID technology in axillary lymph node localization could significantly improve surgical precision in patients with node-positive breast cancer, providing a wire-free alternative to current localization techniques. These innovations could enhance the accuracy, usability, and scope of the LOCalizer™, making it a more adaptable tool for breast cancer surgery.

The use of the effectiveness of non-wired non-ionizing (NWNI) localization technologies has been increasing due to a global shift away from WL. Furthermore, the indications for preoperative localization of non-palpable breast lesions and axillary lymph nodes have expanded, driven not only by the rising incidence of screening-detected breast cancer but also by the de-escalation of breast cancer surgery after neoadjuvant NST and the need for localization in cases of ipsilateral in-breast tumor recurrence following previous BCS [13,14]. Recently, a new paradigm, known as extreme oncoplastic BCS, has been introduced for mastectomy candidates with large multifocal and multicentric tumors or extensive DCIS. This approach has expanded the eligibility for BCS by enabling the application of advanced surgical techniques to tumors that would have previously required mastectomy. Such procedures often necessitate bracketing, utilizing a minimum of two localization active markers to ensure precise tumor excision while preserving breast aesthetics [15].

A recent meta-analysis [16] assessing NWNI techniques in preoperative localization of non-palpable breast lesions, compared to traditional WL, spanned 27 studies and encompassed 2103 procedures. NWNI methods, including magnetic seeds (Magseed, MaMaLoc, MOLLI), radiofrequency RFID tags (LOCalizer™), and infrared reflectors (SAVI SCOUT), demonstrated excellent performance in lesion localization. The overall positive margin rate for NWNI techniques was 12%, indicating their potential to reduce re-excision rates, which were observed at 14%. Compared to WL, NWNI techniques exhibited a lower positive margin rate (12% vs. 17%) and slightly reduced re-excision rates, though the difference was not statistically significant. A recent pooled analysis has demonstrated that wire-free techniques provide statistically significant reductions in re-excision rates and positive margin rates compared to WL [17]. No serious complications were reported, and improved cosmetic outcomes were noted due to more precise lesion localization and minimized removal of healthy tissue.

An additional advantage of NWNI methods is the ability to place the active markers days in advance, enhancing scheduling flexibility and improving patient comfort. Despite these promising findings, further randomized trials are needed to confirm their long-term clinical benefits, cost-effectiveness, and overall impact on breast-conserving surgery outcomes. 

Several barriers, however, hinder the universal adoption of NWNI localization technologies for breast cancer surgery. The high cost of devices, specialized equipment, and training limits accessibility, particularly in resource-limited settings. Implementing NWNI techniques also requires training for surgeons, radiologists, and operating room staff, with resistance to change and workflow adjustments posing challenges. Regulatory approvals and reimbursement issues further impact adoption, as some NWNI devices may not yet be widely approved or covered by healthcare systems and insurers. Additionally, the need for specialized detection systems and probes can create logistical barriers, particularly in centers lacking the necessary infrastructure. While NWNI technologies offer greater scheduling flexibility, integrating them into existing surgical and radiology workflows requires coordination. Surgeon and radiologist preferences also play a role, as many clinicians have extensive experience with WL and may be reluctant to transition unless clear advantages in efficiency and outcomes are demonstrated. Overcoming these barriers will require cost reduction, expanded training programs, regulatory support, and streamlined workflow integration to facilitate the broader adoption of NWNI localization methods. 

## 5. Conclusions

The LOCalizer™ RFID system demonstrated a high accuracy rate for preoperative localization of breast lesions, presenting a viable alternative to WL. This technique improves surgical scheduling flexibility and enhances patient comfort. Comparative studies with other wire-free localization technologies, such as magnetic seeds and radar reflectors, are needed to determine the optimal approach for clinical practice. 

## Figures and Tables

**Table 1:** Primary and secondary objectives and outcomes.

Objectives	Primary/Secondary Objective	Outcomes	Source Data
To assess the accuracy of preoperative RFID tag localization using the LOCalizer™ Hologic system.	Primary	RFID tag placement accuracy was defined as being within 10 mm of the edge of the target lesion. (Benchmark of >95% of RFID tags being within 10 mm of the lesion was modeled on the NHS Breast Screening Programme’s standard, which considers >95% of localization wires within this range as acceptable).	Distance from the RFID tag to the lesion/clip as measured on the post-RFID tag insertion mammograms.
To assess the different types and sizes of lesions being targeted by the RFID tags.	Secondary	Types and size of breast abnormalities localized using the LOCalizer™ device determined through review of patient imaging (including mass lesions, microcalcifications, asymmetric densities, distortions and mammographically occult lesions (post-biopsy marker clips targeted).	Patient records and imaging database.
To evaluate the rates of re-excision following RFID tag placement pre-operatively.	Secondary	The occurrence of re-excision rates through assessment of histological data and reports detailing involvement of surgical margins and the resulting rate of revision surgery.	Anatomo-pathological report on the surgical specimen.
To assess the postoperative specimen pathology radiograph to confirm the presence of the RFID tag.	Secondary	Post-specimen radiographs manually reviewed and assessed following upload to imaging system for presence of RFID tag within the sample.	Postoperative specimen pathology radiograph.

**Table 2:** Types of breast abnormalities localized using the LOCalizer™ device.

Type of Abnormality	Total Number	Range of Size (mm)
Mass lesions	190	5.5–57
Micro-calcifications	54	5–50
Asymmetric densities	15	13–25
Distortions	5	20–25
Mammographically occult (post biopsy marker clips targeted)	33	n/a

## Data Availability

Datasets generated during this study are publicly available in this open access publication without any restrictions.
